# Population pharmacokinetic study in children with vascular anomalies: body weight as a key variable in predicting the initial dose and dosing frequency of sirolimus

**DOI:** 10.3389/fphar.2024.1457614

**Published:** 2024-09-24

**Authors:** Lin Fan, Hong-Li Guo, Yue-Tao Zhao, Yue Li, Wei-Jun Wang, Jian Huang, Ya-Hui Hu, Ji-Jun Zou, Feng Chen

**Affiliations:** ^1^ Pharmaceutical Sciences Research Center, Department of Pharmacy, Children’s Hospital of Nanjing Medical University, Nanjing, China; ^2^ School of Basic Medicine and Clinical Pharmacy, China Pharmaceutical University, Nanjing, China; ^3^ Department of Burns and Plastic Surgery, Children’s Hospital of Nanjing Medical University, Nanjing, China

**Keywords:** sirolimus, population pharmacokinetics, vascular anomalies, children, dosing recommendation

## Abstract

**Background:**

The main challenges faced when using sirolimus in children with vascular anomalies (VAs) still include significant pharmacokinetic (PK) variability, uncertainty in the target concentration range, as well as inconsistencies in initial dosing and dosing frequency. The aim of this study is to establish a new population pharmacokinetic (PPK) model for children with VAs to guide the individualized use of sirolimus.

**Methods:**

A PPK study was performed using data from children with VAs who received sirolimus between July 2017 and April 2022. A nonlinear mixed-effect modeling with a one-compartment model structure was applied. Monte Carlo simulation was employed to propose specific dosing recommendations to achieve the target trough concentrations (*C*_trough_) of 5–15 ng/mL.

**Results:**

In total, 134 blood concentrations from 49 pediatric patients were used to characterize the sirolimus pharmacokinetics. Covariate analysis identified body weight (BW) as a significant factor affecting clearance (*CL*) in the final PPK model. The typical clearance rate and distribution volume, standardized to a BW of 16 kg, were 4.06 L/h (4% relative standard error, RSE) and 155 L (26% RSE), respectively. Optimal dosing regimens were simulated for different BWs. For a twice-daily regimen, the recommended doses were 0.05, 0.06, 0.07, and 0.08 mg/kg/day for BW of <10, 10–20, 20–40, and ≥40 kg, respectively; for a once-daily regimen, the recommended doses were 0.06, 0.07, 0.08, and 0.09 mg/kg/day for BW of <10, 10–30, 30–50, and ≥50 kg, respectively. Notably, sirolimus *C*_trough_ could be maintained between 5–15 ng/mL across various dosing frequencies based on the recommended dosing regimen.

**Conclusion:**

We established a PPK model of sirolimus for children with VAs and proposed an initial dosing strategy. Integrating initial dose and medication frequency recommendations into sirolimus’ guidelines will broaden its clinical options and simplify the clinical management for childhood VAs.

## 1 Introduction

Sirolimus, also known as rapamycin, is an immunosuppressive drug initially approved for use in renal transplantation (FDA, 2024). By blocking downstream protein synthesis and subsequent cell proliferation and angiogenesis through the PI3K/AKT/mTOR pathway (Queisser et al., 2021), sirolimus has shown promising effects in treating vascular anomalies (VAs) including tufted angioma, kaposiform hemangioendothelioma, lymphatic and venous malformations in recent years (Sandbank et al., 2019; Freixo et al., 2020).

Despite the increasingly widespread clinical use of sirolimus, clinical challenges persist, possibly due to its significant pharmacokinetic (PK) variability (Goyal et al., 2013). An 4.5-fold variability in sirolimus clearance (*CL*) was observed among stable renal transplant patients (Zimmerman and Kahan, 1997). Moderate liver impairment caused a 53% decrease in the oral apparent *CL* rate (*CL/F*) of sirolimus (Kovarik et al., 2001), while severe liver impairment patients experience a decrease of up to 67% (Zimmerman et al., 2008). However, these findings are mostly derived from transplant patients, rarely from patients with VAs, and reports in pediatric patients with VAs are rarer. Moreover, it has also been reported that PK parameters of sirolimus varied among races (Zimmerman and Kahan, 1997). In the Asian and Caucasian populations, *ABCB1* C1236T polymorphism had a significant impact on the C_0_/D ratio of sirolimus in Caucasians but nor in Asians (Shao et al., 2020).

Sirolimus has a narrow therapeutic window, typically 8–12 ng/mL or 5–15 ng/mL (Shen et al., 2023; Zhang et al., 2023). It is generally considered that trough concentrations (*C*_trough_) exceeding 15 ng/mL are associated with an increasing risk of sirolimus-induced thrombocytopenia, leukopenia, and hypertriglyceridemia, while concentrations below 5 ng/mL correlate with insufficient therapeutic effects (Kahan et al., 2000). Therefore, therapeutic drug monitoring (TDM) is commonly recommended in clinical practice (FDA, 2024).

Notwithstanding, significant individual differences in the pharmacokinetics, tolerability, and effectiveness of sirolimus treatment persist. Part of the reason for this can be attributed to the only moderate correlation between its steady-state *C*_trough_ and the complete area under the plasma concentration-time curve (Shen et al., 2023). In this case, transitioning from single TDM to model-guided precision dosing becomes particularly necessary. Population pharmacokinetics (PPK) modeling can compensate TDM’s limitations by identifying sources of variability and quantifying the impact of each covariate, providing estimates of PK parameters and their inter-and intra-variability in specific populations (Mould and Upton, 2013). This helps in understanding differences and variations among target populations, thereby assisting in the determination of safe and efficacious drug administration (Chen J. et al., 2023).

Indeed, some researchers have already endeavored to develop PPK models for sirolimus in children with VAs (Mizuno et al., 2017; Wang et al., 2019; Chen et al., 2020; 2021) (Supplementary Table S1). However, these models either suffer from limitations such as small sample sizes (e.g., the studies by Chen and Wang included only 14 to 17 subjects) or inapplicability to the Chinese pediatric population (e.g., Mizuno’s work focused on United States populations). Therefore, a PPK model with a large sample size is warranted to explore the individualized dosing of sirolimus in Chinese children with VAs.

Of note, the initial dose and dosing frequency of sirolimus are also clinical issues that deserve attention. For the treatment of children with VAs, consensus on initial sirolimus dosing is lacking, with reported initial doses ranging from 0.6 mg/m^2^ twice daily to 1.6 mg/m^2^/d and 0.08 mg/kg/d (Sandbank et al., 2019; Maruani et al., 2021; Wiegand et al., 2022). Furthermore, there is no agreement on the frequency of administration. While several studies advocate for twice-daily administration for children due to the shorter half-life of sirolimus compared to adults (Schachter et al., 2004; FDA, 2024), once-daily administration is also prevalent in clinical practice (Schachter et al., 2006; Zhang et al., 2022). Indeed, individualized initial dosing strategy can be achieved through model-based simulations, allowing plasma concentrations to quickly reach the target range, thereby improving tolerability and effectiveness in pediatric patients. This approach has been successfully applied in multiple populations (Dai et al., 2022; Chen L. et al., 2023).

Hence, we are attempting to establish a new PPK model of sirolimus specifically for Chinese children with VAs. Notably, through Monte Carlo simulations, we aimed to develop optimal dosing strategies and provide new insights into individualized sirolimus administration for the treatment of childhood VAs.

## 2 Methods

### 2.1 Patients and data collection

This retrospective study was conducted at the Children’s Hospital of Nanjing Medical University. Pediatric patients diagnosed with VAs who received oral sirolimus treatment and carried out TDM between July 2017 and April 2022 were enrolled. The common initial dosing regimen was 0.08 mg/kg/d, with dosing intervals of either 12 or 24 h. Exclusion criteria included values beyond the detection limit, as well as ongoing serious infections or multiple organ injuries.

The study was approved by the Ethics Committee of the Children’s Hospital of Nanjing Medical University (protocol number: 202206114-1). Written consents were exempted in the ethical approval documents due to the nature of the retrospective study design.

Clinical and laboratory data, including age, sex, body weight (BW), high-density lipoprotein cholesterol (HDL), alanine aminotransferase (ALT), aspartate amino-transferase (AST), total bilirubin (TBIL), direct bilirubin (DBIL), red blood cell count (RBC), hemoglobin (HGB), white blood cell count (WBC), mean corpuscular hemoglobin (MCH), mean corpuscular hemoglobin concentration (MCHC), hematocrit (HCT), albumin (ALB), blood urea nitrogen (BUN), serum creatinine (SCR), cystatin-C (CYSC), and uric acid (UA) were extracted from the hospital’s information system.

### 2.2 Sample analyzing and genotyping

Indeed, the whole blood samples are routinely transported to our laboratory for monitoring plasma sirolimus levels in children with VAs. Briefly, the whole blood samples (1–2 mL) were collected into the EDTA K2 anticoagulant tube for routine TDM at least 7 days after the start of sirolimus therapy, specifically 30 min before the next maintenance dose. After concentration measurement, the left-over samples were separated by centrifugation for collecting plasma and blood cell sediment and then stored at −80°C for subsequent analysis. Enzyme multiplied immunoassay technique (Emit^®^ 2000; SIEMENS, Munich, Germany) with the calibration range of 3.5–30 ng/mL was employed for the quantitative analysis for sirolimus (Zhao et al., 2022). To ensure accuracy and precision, three levels of quality control samples with a deviation of ± 15% were utilized. The deviations of quality control samples over the period of clinical sample collection and detection were from −13.2% to 14.8%.

The blood samples used for genotyping were from TDM residual samples. DNA was extracted by using a DNA kit (Zhongkebio Med Technol, Nanjing, China). The analysis was conducted by BGI Technologies (Shenzhen, China) using the Agena MassARRAY platform 4.0 with iPLEX gold chemistry (Agena Bioscience, Inc., CA, United States). More genotyping data can be found in Supplementary Table S2. The Hardy-Weinberg equilibrium was evaluated using the chi-square goodness-of-fit test to scrutinize deviations in allele and genotype frequencies across different genes.

### 2.3 PPK modeling

Parameter estimation was carried out using a nonlinear mixed effects model program (NONMEM, v7.3.0, Icon Inc., PA, United States) and a first-order conditional estimation method with interaction (FOCE-I). Data processing and visualization were performed with R (v4.3.1) and Prism 9 (v9.5.0). Pirana software (Version 2.9.7) served as the workbench of NONMEM.

#### 2.3.1 Base model

A one-compartment model with first-order elimination was suitable to describe sirolimus’ pharmacokinetics since all the concentrations in this study were trough concentrations. The apparent volume of distribution (*V/F*) and *CL/F* were described. Due to the absence of observations during the absorption phase, absorption rate constant (*K*a) was established at 0.485 h^−1^ according to previously reports in the literature (Wang et al., 2020; Chen et al., 2022).

Exponential model was chosen to evaluate inter-individual variability of the PK parameters and was described as Equation 1.

$$Pi=TV\left(P\right)\times e^{\eta i}$$ (1)

where *Pi* represents the individual parameter value; *TV(P)* represents the typical individual parameter value; and *ηi* represents the variability between subjects.

Additive (Equation 2), exponential (Equation 3), and mixed error (Equation 4) models were evaluated to describe the residual variance.

Y=IPRED+ε (2)

$$Y=IPRED\times e^{\varepsilon }$$ (3)

$$Y=IPRED\times \left(1+\varepsilon 1\right)+\varepsilon 2$$ (4)

where *Y* represents the individual observation; *IPRED* represents the individual prediction; and *ε* represents a randomly distributed variable.

#### 2.3.2 Covariate model

Since age and weight have been wildly recognized in previous studies as important variables influencing the PK parameters of sirolimus in children (Sabo et al., 2021; Li et al., 2022), these two covariates were initially evaluated in a series of size and maturation models (Anderson and Holford, 2008; Holford et al., 2013). The maturation model that achieved the smallest objective function values (OFV) was further developed as the intermediate model. The general maturation model (Equation 5) was as follows:

$$Pi=TV\left(P\right)\times {\left(\frac{BW}{{BW}_{median}}\right)}^{m}\times MF$$ (5)

where m is the exponent of weight and MF is a maturation factor with the following five forms:

Model I: simple exponential model (Equations 6, 7), exponents m and n are estimated

$$CL/F=TV\left(CL\right)\times {\left(\frac{BW}{{BW}_{median}}\right)}^{m}$$ (6)

$$V/F=TV\left(V\right)\times {\left(\frac{BW}{{BW}_{median}}\right)}^{n}$$ (7)

Model II: fixed allometric exponent model (Equations 8, 9)

$$CL/F=TV\left(CL\right)\times {\left(\frac{BW}{{BW}_{median}}\right)}^{0.75}$$ (8)

$$V/F=TV\left(V\right)\times \left(\frac{BW}{{BW}_{median}}\right)$$ (9)

Model III: sigmoid maturation model (Equation 10), where TM50 is the age at which *CL* maturation reaches half of the adult’s *CL*, and Hill is the slope parameter for the sigmoid E_max_ maturation model

$$CL/F=TV\left(CL\right)\times {\left(\frac{BW}{{BW}_{median}}\right)}^{0.75}\times MF,MF=\frac{1}{1+{\left(\frac{Age}{TM50}\right)}^{Hill}}$$ (10)

Model IV: weight dependent exponent model (Equation 11)

$$CL/F=TV\left(CL\right)\times {\left(\frac{BW}{{BW}_{median}}\right)}^{m},m=k_{0}-\frac{K_{\max}\times {BW}^{Hill}}{{K_{50}}^{Hill}+{BW}^{Hill}}$$ (11)

Model V: age dependent exponent model (Equation 12)

$$CL/F=TV\left(CL\right)\times {\left(\frac{BW}{{BW}_{median}}\right)}^{m},m=k_{0}-\frac{K_{\max}\times {Age}^{Hill}}{{K_{50}}^{Hill}+{Age}^{Hill}}$$ (12)

In models IV and V, *k*_0_ is defined as an exponent at a theoretical weight of 0 or at an age of 0 years, respectively. *K*_max_ represents the maximum reduction of the exponent. The Hill coefficient determines the steepness of the sigmoid decline. *k*_50_ indicates the weight (in Model IV) or age (in Model V) at which there is a 50% decrease relative to the maximum decrease.

Other potential covariates including ALT, AST, HDL, TBIL, DBIL, RBC, WBC, HGB, MCH, MCHC, HCT, ALB, SCR, BUN, UA, CYSC, single nucleotide polymorphisms (SNPs) of *CYP3A4* (i.e., rs4646437 and rs2242480), SNPs of *CYP3A5* (rs776746), SNPs of *mTOR* (i.e., rs1883965, rs2076655 and rs2300095), SNPs of *ABCB1* (rs1128503), SNPs of *ABCC2* (rs717620), SNPs of *CYP3A7* (i.e., rs12360, rs10211, and rs2257401), SNPs of *POR* (rs10578680), SNPs of *IL10* (rs1800896), SNPs of *IL18* (rs5744247), SNPs of *SUM04* (rs237024), SNPs of *NR1I2* (rs3814055 and rs6785049), and SNPs of *TCF7L2* (rs7903146) were investigated. Linear model, power model, and exponential model (Equations 13–15, respectively) were used to describe the continuous covariates, while the additive model and proportional model (Equations 16, 17, respectively) were used to describe categorical covariates.

$$P_{i}=TV\left(P\right)+\theta \times \left(\frac{COV}{{COV}_{median}}\right)$$ (13)

$$P_{i}=TV\left(P\right)\times {\left(\frac{COV}{{COV}_{median}}\right)}^{\theta }$$ (14)

$$P_{i}=TV\left(P\right)\times e^{\theta \times \left(\frac{COV}{{COV}_{median}}\right)}$$ (15)

$$P_{i}=TV\left(P\right)+\theta \times COVF_{i}$$ (16)

$$P_{i}=TV\left(P\right)\times \left(1+\theta \times COVF_{i}\right)$$ (17)

where θ is the estimate of the effect of the covariate on the parameter.

The covariates were screened in a stepwise way with forward inclusion and backward exclusion. A decrease in the OFV of at least 3.84 (*P < 0.05, df = 1*) and an increase in the OFV of at least 10.84 (*P < 0.001, df = 1)* were considered as the standard to include or to retain significant covariates, respectively. Notably, if a significant correlation was observed between covariates, only one was included in the subsequent modeling (Bonate, 1999). Additionally, the accuracy and physiological rationality of the parameters were considered throughout the stepwise process.

#### 2.3.3 Model evaluation

The performance of the final model was visually assessed using goodness-of-fit (GOF) plots and normalized prediction distribution error (NPDE). The statistical tests of NPDE were conducted through the NPDE R package (Comets et al., 2008). The precision of model parameter estimates was assessed based on the standard errors calculated using the covariance matrix method, which is the default setting in NONMEM (R^−1^ SR^−1^). The predictive capability of the final model was further validated by using numerical predictive check (NPC) and visual predictive check (VPC), each performed with 1,000 simulations. Additionally, bootstrap with repetition of 1,000 runs was applied to evaluate the stability and reliability of the final estimates. Finally, the precision of the model was estimated by the mean prediction error (MPE), mean absolute prediction error (MAPE), mean relative prediction error (MPE%), mean relative absolute prediction error (MAPE%), root mean squared prediction error (RMSE), and composite indices *F*_20_ and *F*_30_, which represent the percentage of prediction errors within ± 20% and ± 30%, respectively.

Equations 18–21 are as follows:

$$PE\%=\frac{pred-obs}{obs}\times 100\%$$ (18)

$$MPE=\frac{1}{N}\sum_{1}^{N}\left({pred}_{i}-{obs}_{i}\right);MPE\%=\frac{1}{N}\sum_{1}^{N}\left(\frac{{pred}_{i}-{obs}_{i}}{{obs}_{i}}\right)\times 100\%$$ (19)

$$MAPE=\frac{1}{N}\sum_{1}^{N}\left|{pred}_{i}-{obs}_{i}\right|;\;MAPE\%=\frac{1}{N}\sum_{1}^{N}\left(\frac{\left|{pred}_{i}-{obs}_{i}\right|}{{obs}_{i}}\right)\times 100\%$$ (20)

$$RMSE=\sqrt{\frac{1}{N}\sum_{1}^{N}{\left({pred}_{i}-{obs}_{i}\right)}^{2}}$$ (21)

#### 2.3.4 Simulations

Monte Carlo simulations were carried out using NONMEM (v7.3.0, Icon Inc., PA, United States) to identify the optimal initial sirolimus dose to achieve its targeted *C*_trough_ of 5–15 ng/mL (Lackner et al., 2015; Ozeki et al., 2019; Sandbank et al., 2019). A total of 1,000 simulations were conducted for each clinical scenario. The dosing regimens ranged from 0.04 to 0.12 mg/kg/d at 0.01 mg interval were evaluated to identify the optimal dose in pediatric patients at different BW groups (<10, 10–20, 20–30, 30–40, 40–50, and ≥50 kg), respectively. We also performed a simulation for a virtual 25-kg pediatric patient with VAs (0.07 mg/kg/d, monotherapy) to generate a concentration-time curve, thereby illustrating the changes in plasma sirolimus concentration under different dose intervals (12 h or 24 h) based on clinical dosing practice. The evaluation criteria were the probabilities of achieving concentrations within the target range.

## 3 Result

### 3.1 Subjects

The model included 49 children with VAs, of whom 24 were males. A total of 134 concentrations were collected. The median BW of all subjects was 16 kg, ranging from 3.3 to 65 kg. The dose of sirolimus was ranged from 0.018 to 0.152 mg/kg/d. Table 1 summarizes the main demographic and clinical characteristics. The detailed information of subject characteristics obtained for subsequent PPK modeling is shown in Supplementary Table S3.

**TABLE 1:** Demographic, laboratory, and genotype data of enrolled subjects.

Characteristic	Median (range)
Demographic	
Sex, male/female	24/25
Age, y	3.5 (0.08–12)
WT, kg	16 (3.3–65)
Laboratory parameter	
RBC (10^12^/L)	4.64 (2.73–5.9)
WBC (10^9^/L)	8 (4.33–17.14)
HGB (g/L)	122 (77–172)
MCH (pg)	26.7 (19.4–30.6)
MCHC (g/L)	330 (213–361)
HCT (%)	36.8 (24.2–49.4)
ALB (g/L)	44.8 (37.2–52.2)
ALT (U/L)	12 (5–34)
AST (U/L)	27 (17–52)
HDL (mmol/L)	1.38 (0.6–2.15)
TBIL (μmol/L)	5.5 (1.6–16)
DBIL (μmol/L)	1.97 (0–7.3)
Genotype	
*CYP3A4*	
rs4646437 (%) GG/GA/AA	34/12/3
rs2242480 (%) CC/CT/TT	28/15/6
*CYP3A5*	
rs776746 (%) TT/CT/CC	5/22/22

Abbreviations: BW, total body weight; RBC, red blood cell count; WBC, white blood cell count; HGB, hemoglobin; MCH, mean corpuscular hemoglobin; MCHC, mean corpuscular hemoglobin concentration; HCT, hematocrit; ALB, albumin; ALT, alanine aminotransferase; AST, aspartate amino transferase; HDL, high-density lipoprotein cholesterol; TBIL, total bilirubin; DBIL, direct bilirubin.

### 3.2 Model development

Since only *C*_trough_ were applied in the study, the inter-individual variability of *V/F* was not estimated. The residual variability was best described by an additive error model (Equation 2). Among five different maturation models, the OFV values of Model I was the lowest (Supplementary Table S4). Therefore, the simple exponential model was selected for further covariate screening. However, no other covariates showed significant influence on sirolimus *CL/F* during forward inclusion and backward exclusion phase. Details of the covariate screening process are shown in Supplementary Table S4. The finial model (Equations 22, 23)was as follows:

$$CL/F=4.06\times {\left(\frac{BW}{16}\right)}^{1.23}$$ (22)

$$V/F=155\times {\left(\frac{BW}{16}\right)}^{1.62}$$ (23)

### 3.3 Model evaluation

**FIGURE 1:**
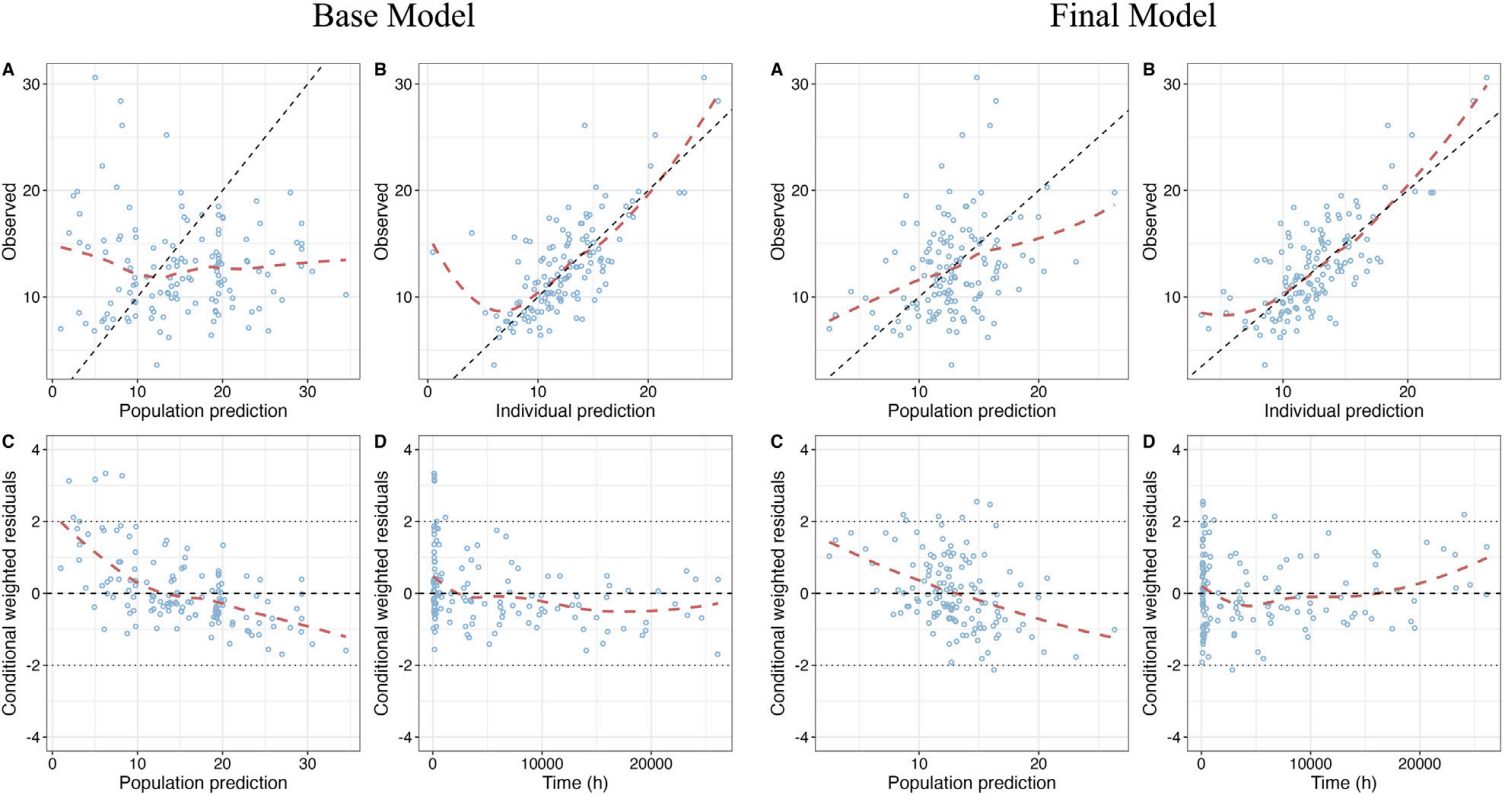
Goodness-of-fit (GOF) plots of base model and final model. **(A)** Dependent variable (DV) vs. Population prediction (PRED); **(B)** DV vs. Individual prediction (IPRED); **(C)** CWRES vs. PRED; **(D)** Conditional weighted residuals (CWRES) vs. Time after first dose.

GOF plots of the base model and the final model are shown in Figure 1. The prediction performances of the final model were significantly improved compared to the base model with no obvious bias or significant trends that were deviated from y = x or y = 0. Most of the conditional weighted residuals (CWRES) were randomly distributed around zero line and most of the residuals were within ± 2.

**FIGURE 2:**
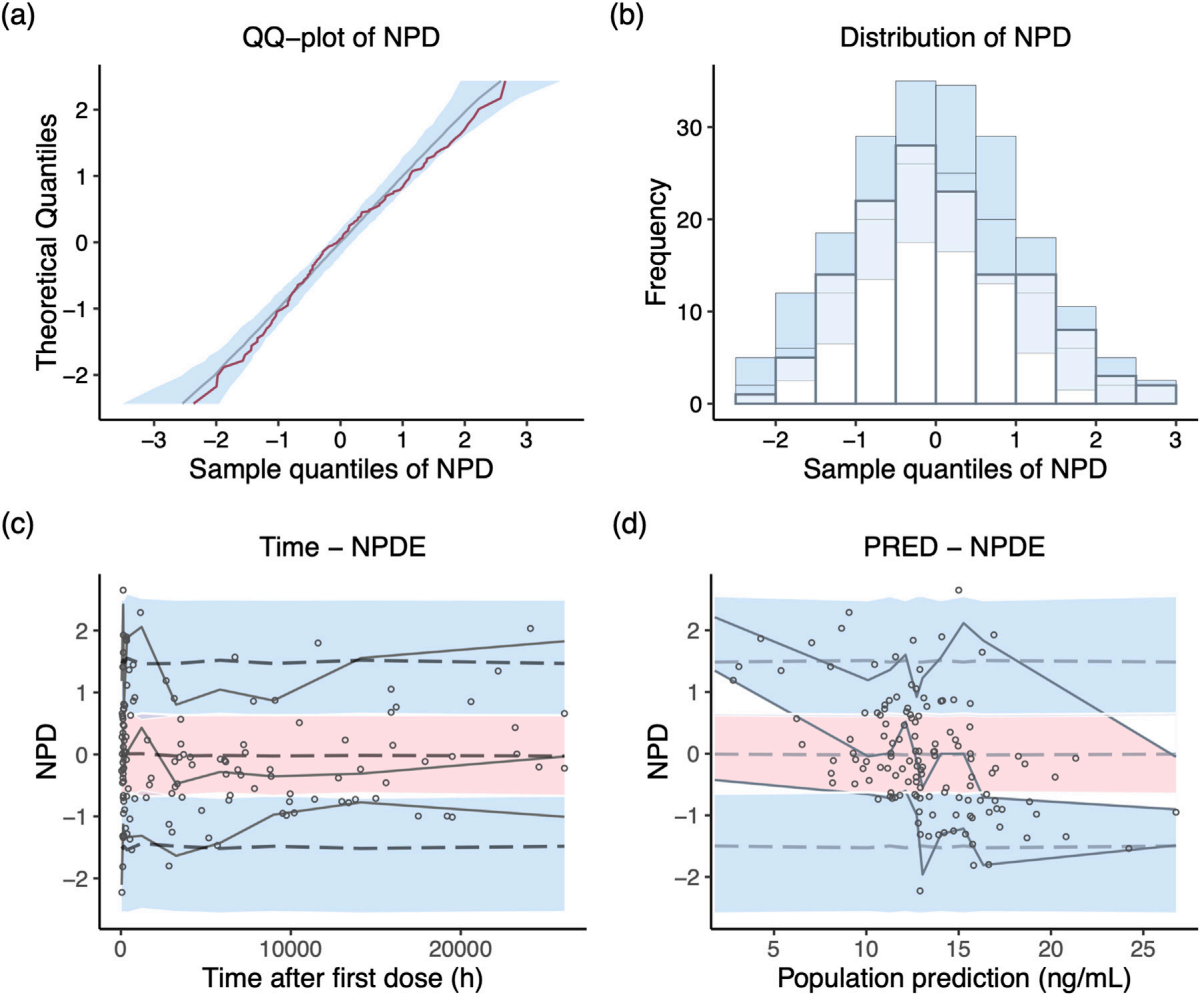
Normalized prediction distribution (NPD) plots of the final model. **(A)** Q-Q plot of the distribution of the NPD vs. theoretical normal distribution; **(B)** histogram of the distribution of the NPD; **(C)** NPD vs. time after first dose; **(D)** NPD vs. population prediction.

**FIGURE 3:**
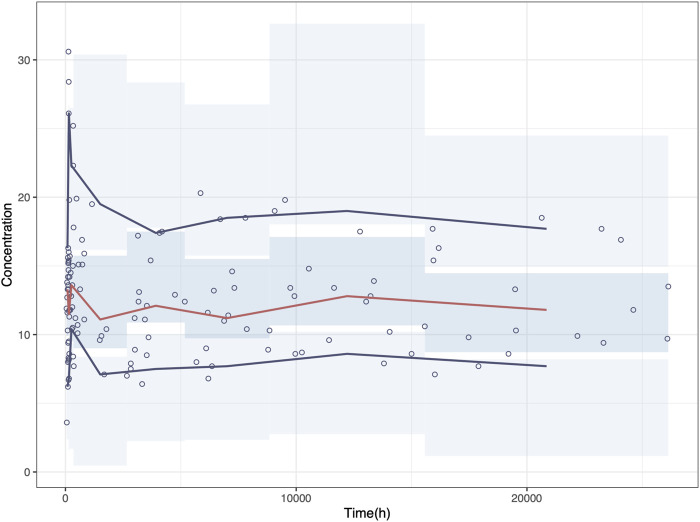
Visual predictive check (VPC) plot of the final model. Circles represent the observed concentrations. The red solid line represents the median of the prediction-corrected concentrations. The blue solid lines represent the 2.5% and 97.5% of the prediction-corrected concentrations, respectively. The shaded areas represent the 95% confidence interval of each line.

NPD plot is shown in Figure 2. No trends were observed in the scatterplots and the statistical tests of NPDE showed a normal distribution with a theoretical mean of 0.035 and variance of 1.036. The VPC plot, shown in Figure 3, indicated good predictive performance of the final model, as most of the observations were included in the 95% prediction intervals derived from the simulation data. NPC result, as a numerical statistical supplement to VPC, is shown in Supplementary Table S5.

The parameter estimates of the final model and bootstrap analysis are presented in Table 2. The median values of bootstrap estimation were close to the respective values of the final model, with all the biases being less than 5%, and all the final model estimates fell within the 95% CI of the bootstrap estimates. The success rate of 1,000 times bootstrap runs was 96.8%, indicating that the model was stable and reliable. As presented in Table 3, the prediction errors were small in final model with MPE% 3.51% ≤ ±20%, MAPE% 2.42% ≤ 30%, F_20_ 38.81% ≥ 35%, and F_30_ 55.22% ≥ 50%, respectively.

**TABLE 2:** Final estimates and bootstrap analysis of final model.

Parameter	Final model		Bootstrap		Bias (%)
	Estimate	RSE% [%shrinkage]	Median	95%CI	
*CL/F* (L/h)	4.06	4	4.03	3.58–4.39	−0.84
*V/F* (L)	155	26	160	60–246	3.00
m	1.23	8	1.22	0.98–1.43	−0.42
n	1.62	13	1.59	1.09–2.07	−1.56
Ka (h^−1^)	0.485	fixed	0.485	—	—
ω_CL_ (%)	25.94	32 [17]	24.90	14.58–33.97	−4.04
σ (ng/mL)	3.41	14 [13]	3.36	2.83–3.88	−1.34

Bias (%) = (Median - Estimate)/Estimate × 100%.

Abbreviations: *CL/F*, apparent clearance; *V/F*, apparent distribution volume; ω_cl_, inter-individual variability in *CL*; σ, residual variability; RSE%, relative standard error; 95% CI, 95% confidence interval.

**TABLE 3:** The prediction performance of the final model.

MPE	MPE%	MAPE	MAPE%	RMSE	*F*_20_%^a^	*F*_30_%^a^	*F*_*i*20_%^b^	*F*_*i*30_%^b^
−0.19	3.51	2.42	20.95	2.96	38.81	55.22	59.7	78.36

Abbreviations: MPE, mean prediction error; MAPE, mean absolute prediction error; RMSE, root mean square error.

^a^ PE% between ± 20% and ± 30% based on population prediction.

^b^ PE% between ± 20% and ± 30% based on individual prediction.

### 3.4 Simulation and optimization

**FIGURE 4:**
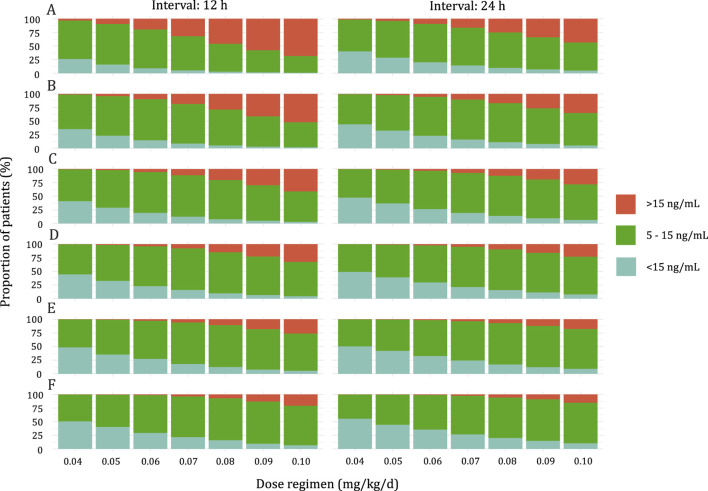
Stacked bar graph of the probability of Sirolimus *C*_trough_ at steady state above range (>15 ng/mL), below range (<5 ng/mL), or within range (5–15 ng/mL). **(A)** Pediatric patients weighing <10 kg. **(B)** Pediatric patients weighing 10–20 kg. **(C)** Pediatric patients weighing 20–30 kg. **(D)** Pediatric patients weighing 30–40 kg. **(E)** Pediatric patients weighing 40–50 kg. **(F)** Pediatric patients weighing ≥50 kg.

Simulations were conducted to determine the optimal initial dose for pediatric patients with varying weight according to the target trough concentration of 5–15 ng/mL. The results of the simulation are presented in Figure 4. The recommended doses are shown in Table 4. With an increase in BW, a higher BW-normalized sirolimus dose was required.

**TABLE 4:** Optimal dosing regimens for targeted *C*_trough_ between 5 ng/mL and 15 ng/mL.

Body weight (kg)	Dose (mg/kg/d)	% *C*_trough_		
		<5 ng/mL	5–15 ng/mL	>15 ng/mL
Interval: 12 h				
<10	0.05	13.76	76.27	9.97
10–20	0.06	12.64	77.26	10.10
20–30	0.07	10.87	77.33	11.80
30–40	0.07	13.72	78.34	7.94
40–50	0.08	10.33	78.80	10.87
≥50	0.08	13.65	78.61	7.74
Interval: 24 h				
<10	0.06	18.39	72.83	8.78
10–20	0.07	14.90	75.05	10.04
20–30	0.07	12.74	75.38	11.88
30–40	0.08	14.55	76.32	9.14
40–50	0.08	14.42	78.18	7.40
≥50	0.09	13.73	77.24	9.03

**FIGURE 5:**
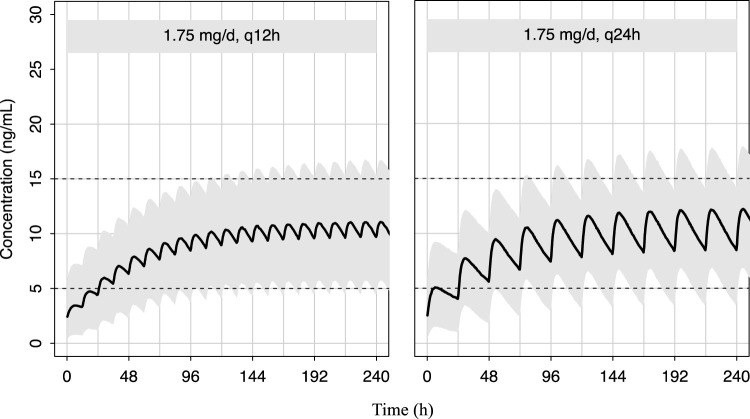
Predicted distribution of sirolimus concentration-time profiles in 1,000 simulated pediatric patients weighing 25 kg. The solid lines depict the median of the simulated data, and the shaded areas represent the 80% prediction interval for the simulated individuals and the dashed lines indicate the lower and upper boundaries of the target range of 5–15 ng/mL.

As shown in Figure 5, the predicted sirolimus concentration-time profiles in children with BW of 25 kg under the recommended dose of 0.07 mg/kg/d were simulated. Under both dosing intervals, steady-state concentrations achieved within 7–8 days. Although the plasma concentration of sirolimus exhibited reduced fluctuation with a dosing interval of 12 h, it remained within the target concentration range even when the dosing interval was extended to 24 h. This suggests that sirolimus maintains therapeutic levels despite alterations in dosing frequency.

## 4 Discussion

The considerable inter- and intra-individual variability in pharmacokinetics, its narrow therapeutic range, and the correlation between dosage and adverse reactions of sirolimus highlight the pressing need for establishing a rational initial dosing regimen for treating children with VAs (Shen et al., 2023). By establishing a PPK model for sirolimus in children with VAs, we proposed a detailed dosing strategy for pediatric patients based on their BWs. To the best of our knowledge, this study is the largest PPK modeling study on Chinese children with VAs.

In this study, BW as an important covariate was found in the final model. Indeed, we observed a nonlinear increase in *CL/F* with BW gain, which was line with prior PPK studies on sirolimus in children with VAs (Mizuno et al., 2017; Chen et al., 2020; 2021) (Supplementary Table S1). Children are in the growth and development stage, which is the main physiological feature that distinguishes them from adults. It has been widely known that drug elimination in children mainly increases with their BW and age (Bartelink et al., 2006; Anderson and Holford, 2008; Dai et al., 2022). To assess the impact of BW and age on the PK parameters of sirolimus, five maturation models were examined, among which model I using allometric scaling of BW demonstrated the lowest OFV. Due to the significant correlation between age and BW (correlation coefficient, r = 0.86), age was excluded in the subsequent covariate inclusion process to avoid collinearity and instability in parameter estimation. Indeed, apart from BW, the previous models’ evaluation has included covariates like age (postmenopausal age), sex, ALT, and *CYP3A5* gene polymorphism (Supplementary Table S3). However, all these covariates failed to be included in our study. Of note, since BW was the only covariate ultimately included, this model could be more widely applied in clinical practice.

Apart from BW, we explored other potential factors influencing sirolimus pharmacokinetics, such as liver function, red blood cells, and lipoproteins (McCune et al., 2016; Hartinger et al., 2022). Sirolimus is primarily distributed in red blood cells and shows concentration-dependent binding to lipoproteins in the whole blood compartment (Stenton et al., 2005). It undergoes major metabolism in the liver. Surprisingly, none of these factors demonstrated a significant influence on its *CL/F* and were therefore not included in the final model. This exclusion may be attributed to the insufficient number of patients with liver impairment in our dataset, as well as the fluctuation of blood cells and lipoproteins within normal ranges.

Sirolimus is metabolized by CYP3A4, CYP3A5, and CYP2C8, with CYP3A4 playing the most significant role, followed by CYP3A5 and then by CYP2C8 (Emoto et al., 2013; 2015). It is also a substrate of P-glycoprotein (Moes et al., 2015). However, there is still controversy regarding the impact of *CYP3A4* and *CYP3A5* gene polymorphisms on sirolimus metabolism. Studies have indicated that specific SNPs of *CYP3A4* (*1G and *1B) and *CYP3A5* (*1 and *3) can affect the metabolic activity and oral *CL/F* of sirolimus (Anglicheau et al., 2005; Le Meur et al., 2006). However, other studies have found that SNPs of *CYP3A4* (*22) and *CYP3A5* (*3) are not significantly correlated with sirolimus dose, *C*_trough_, and *C*_trough_/dose ratio in kidney transplant patients (Woillard et al., 2013).

In our modeling, we failed to include the genetic polymorphisms of *CYP3A4* and *CYP3A5* as covariates. This could be attributed to the wide age distribution of our study population (0.08–12 years old), which may lead to varying levels of maturity of CYP3A protein at different developmental stages (de Wildt et al., 1999; Lang et al., 2021). For example, CYP3A4 activity increases rapidly after birth but only reaches about half of adult levels at 6–12 months. Similarly, there are significant individual differences in CYP3A5 expression and activity at various developmental stages (de Wildt et al., 1999). The variation in enzyme activity at different developmental stages may contribute to the model’s inability to accurately estimate the impact of genetic polymorphism. In addition, *CYP3A4* polymorphisms contribute only to a minor extent or only in relatively rare cases to the interindividual differences of the CYP3A4 phenotype (Werk and Cascorbi, 2014). Impressively, previous study by Wang et al. (2020) also failed to incorporate *CYP3A4* and *CYP3A5* polymorphisms into their PPK models of sirolimus in children with tuberous sclerosis.

In clinical practice, common medication regimens for pediatric patients with VAs involve both twice-daily and once-daily administrations. Simulations based on the recommended optimal dosage demonstrate a higher probability of reaching the target concentration twice daily compared to the once-daily regimen across varying BWs (Table 4). However, statistical analysis reveals no significant difference between the two regimens (P = 0.053).

Medication adherence poses considerable challenges within the pediatric population. Studies indicate a medication adherence rate of only 50%–60% among pediatric patients with chronic illnesses (El-Rachidi et al., 2017). Low adherence elevates the risks associated with medication usage and contributes to disease progression. It has been reported that medication adherence is related to administration frequency, with higher frequencies correlating with decreased adherence rates (Bender, 2002; Kardas et al., 2013). Thus, we proposed that patients who can tolerate adverse reactions from a single dose consider a once-daily dosing regimen. This approach may minimize dosing frequency, potentially reduce inconvenience for pediatric patients, and enhancing overall patient adherence.

Our research has several limitations. Firstly, this is a retrospective study, despite our efforts to encompass an extensive array of covariates, certain potential influencing factors, such as the impact of food on sirolimus metabolism (Mizuno et al., 2019), remain beyond our inclusion. Additionally, important covariates like liver function and genetic polymorphisms were failed to be included in the model, which might contribute to an incomplete explanation of the inter-individual variability in the *CL/F* of sirolimus. Notably, the inclusion of BW only explained 58.67% of this variability. Secondly, *K*a was fixed at 0.485 h^−1^ due to a lack of information on the absorption phase. Previous studies have reported a wide range of estimated *K*a from 0.0535 to 2.77 h^−1^ in kidney transplant patients and blood and marrow transplant patients (Ferron et al., 1997; Goyal et al., 2013). However, the differences in *K*a across studies could not be clearly explained (Methaneethorn et al., 2022). Further studies in this field are warranted. The *K*a used in our study was derived from several previous PPK studies on sirolimus (Chen et al., 2020; 2021; Wang et al., 2020), which were also performed in Chinese children with VAs. Nevertheless, it must be acknowledged that different *K*a values can have a certain impact on the PK parameters estimation. Therefore, we recommend exercising caution when applying our model to other racial groups. Thirdly, while the sample size has been augmented, the rarity of VAs as a disease restricts the available pool of participants, thereby resulting in a relatively modest sample size. Consequently, there remains a compelling necessity for large-scale investigations to enhance our understanding of the population’s characteristics comprehensively. Fourthly, it is important to highlight that while many studies, including Italian guideline for VAs (Stillo et al., 2022), suggested a target C_trough_ range of 5–15 ng/mL for symptomatic, progressive, and refractory cystic lymphatic malformations, there remains no consensus on the appropriate C_trough_ range for the broader VAs population. Various prospective clinical studies (Ji et al., 2021; Maruani et al., 2021; Harbers et al., 2023; Seront et al., 2023) have proposed differing target ranges, such as 10–15 ng/mL, 4–10 ng/mL, and 4–12 ng/mL. Therefore, further prospective randomized controlled trials are required to establish the optimal therapeutic target for sirolimus in this population. Lastly, it is important to note that our study did not determine recommended doses through pharmacokinetic-pharmacodynamic (PK-PD) analyses but rather relied solely on the *C*_trough_ of sirolimus mainly because we did not have access to matching PD data. Therefore, further clinical research is warranted to validate the recommended dosing regimens and their impact on treating children with VAs.

## 5 Conclusion

In conclusion, this study has successfully developed and validated a PPK model of sirolimus for children with VAs. Integrating initial dose and medication frequency recommendations into sirolimus guidelines will broaden its clinical options and simplify the clinical management of children with VAs.

### 5.1 Study highlights

#### 5.1.1 What is the current knowledge on the topic?

The main challenges faced when using sirolimus in children with VAs still include significant pharmacokinetic (PK) variability as well as inconsistencies in initial dosing and dosing frequency.

#### 5.1.2 What question did this study address?

Is it possible to describe the inter- and intra-individual variability of sirolimus via PPK model, and then simulate initial dosing and administration frequency through the model?

#### 5.1.3 What does this study add to our knowledge?

This is to date the biggest PPK study on Chinese pediatric patients with VAs. It supplements the sirolimus PK characteristics and provides recommended initial dosing regimens based on BW. This study suggests that sirolimus maintains therapeutic levels despite alterations in dosing frequency.

#### 5.1.4 How might this change drug discovery, development, and/or therapeutics?

Integrating initial dose and medication frequency recommendations into sirolimus guidelines will broaden its clinical options and simplify the clinical management of children with VAs.

## Data Availability

The datasets presented in this study can be found in online repositories. The names of the repository/repositories and accession number(s) can be found in the article/Supplementary Material.

## References

[B1] Anderson B. J. Holford N. H. G. (2008). Mechanism-based concepts of size and maturity in pharmacokinetics. Annu. Rev. Pharmacol. Toxicol. 48, 303–332. 10.1146/annurev.pharmtox.48.113006.094708 17914927

[B2] Anglicheau D. Le Corre D. Lechaton S. Laurent-Puig P. Kreis H. Beaune P. (2005). Consequences of genetic polymorphisms for sirolimus requirements after renal transplant in patients on primary sirolimus therapy. Am. J. Transplant. Official J. Am. Soc. Transplant. Am. Soc. Transpl. Surg. 5, 595–603. 10.1111/j.1600-6143.2005.00745.x 15707415

[B3] Bartelink I. H. Rademaker C. M. A. Schobben A. F. A. M. van den Anker J. N. (2006). Guidelines on paediatric dosing on the basis of developmental physiology and pharmacokinetic considerations. Clin. Pharmacokinet. 45, 1077–1097. 10.2165/00003088-200645110-00003 17048973

[B4] Bender B. G. (2002). Overcoming barriers to nonadherence in asthma treatment. J. Allergy Clin. Immunol. 109, S554–S559. 10.1067/mai.2002.124570 12063512

[B5] Bonate P. L. (1999). The effect of collinearity on parameter estimates in nonlinear mixed effect models. Pharm. Res. 16, 709–717. 10.1023/a:1018828709196 10350015

[B6] Chen J. Huang X. Yu L. Li J. Yang R. Li L. (2023a). Vancomycin population pharmacokinetics analysis in Chinese paediatric patients with varying degrees of renal function and ages: development of new practical dosing recommendations. J. Antimicrob. Chemother. 78, 2037–2051. 10.1093/jac/dkad202 37379498 PMC10393882

[B7] Chen L. Krekels E. H. J. Heijnen A. R. Knibbe C. A. J. Brüggemann R. J. (2023b). An integrated population pharmacokinetic analysis for posaconazole oral suspension, delayed-release tablet, and intravenous infusion in healthy volunteers. Drugs 83, 75–86. 10.1007/s40265-022-01819-8 36607589

[B8] Chen X. Wang D. Wang G. Huang Y. Yu X. Lu J. (2021). Optimization of initial dose regimen for sirolimus in pediatric patients with lymphangioma. Front. Pharmacol. 12, 668952. 10.3389/fphar.2021.668952 34819851 PMC8606893

[B9] Chen X. Wang D.-D. Xu H. Li Z.-P. (2020). Initial dose recommendation for sirolimus in paediatric kaposiform haemangioendothelioma patients based on population pharmacokinetics and pharmacogenomics. J. Int. Med. Res. 48, 300060520947627. 10.1177/0300060520947627 32815764 PMC7444137

[B10] Chen X. Wang J. Lan J. Ge X. Xu H. Zhang Y. (2022). Initial sirolimus dosage recommendations for pediatric patients with PIK3CD mutation-related immunodeficiency disease. Front. Pharmacol. 13, 919487. 10.3389/fphar.2022.919487 36188573 PMC9515533

[B11] Comets E. Brendel K. Mentré F. (2008). Computing normalised prediction distribution errors to evaluate nonlinear mixed-effect models: the npde add-on package for R. Comput. Methods Programs Biomed. 90, 154–166. 10.1016/j.cmpb.2007.12.002 18215437

[B12] Dai H.-R. Liu Y. Lu K.-Y. He X. Guo H.-L. Hu Y.-H. (2022). Population pharmacokinetic modeling of caffeine in preterm infants with apnea of prematurity: new findings from concomitant erythromycin and AHR genetic polymorphisms. Pharmacol. Res. 184, 106416. 10.1016/j.phrs.2022.106416 36029933

[B13] de Wildt S. N. Kearns G. L. Leeder J. S. van den Anker J. N. (1999). Cytochrome P450 3A: ontogeny and drug disposition. Clin. Pharmacokinet. 37, 485–505. 10.2165/00003088-199937060-00004 10628899

[B14] El-Rachidi S. LaRochelle J. M. Morgan J. A. (2017). Pharmacists and pediatric medication adherence: bridging the gap. Hosp. Pharm. 52, 124–131. 10.1310/hpj5202-124 28321139 PMC5345910

[B15] Emoto C. Fukuda T. Cox S. Christians U. Vinks A. A. (2013). Development of a physiologically-based pharmacokinetic model for sirolimus: predicting bioavailability based on intestinal CYP3A content. Cpt Pharmacometrics Syst. Pharmacol. 2, e59. 10.1038/psp.2013.33 23884207 PMC3731827

[B16] Emoto C. Fukuda T. Venkatasubramanian R. Vinks A. A. (2015). The impact of CYP3A5*3 polymorphism on sirolimus pharmacokinetics: insights from predictions with a physiologically-based pharmacokinetic model. Br. J. Clin. Pharmacol. 80, 1438–1446. 10.1111/bcp.12743 26256674 PMC4693485

[B17] FDA (2024). Rapamune Sirolimus product information. Available at: https://www.accessdata.fda.gov/drugsatfda_docs/label/2022/021083s069s070,021110s087s088lbl.pdf (Accessed June 5, 2024).

[B18] Ferron G. M. Mishina E. V. Zimmerman J. J. Jusko W. J. (1997). Population pharmacokinetics of sirolimus in kidney transplant patients. Clin. Pharmacol. Ther. 61, 416–428. 10.1016/S0009-9236(97)90192-2 9129559

[B19] Freixo C. Ferreira V. Martins J. Almeida R. Caldeira D. Rosa M. (2020). Efficacy and safety of sirolimus in the treatment of vascular anomalies: a systematic review. J. Vasc. Surg. 71, 318–327. 10.1016/j.jvs.2019.06.217 31676179

[B20] Goyal R. K. Han K. Wall D. A. Pulsipher M. A. Bunin N. Grupp S. A. (2013). Sirolimus pharmacokinetics in early postmyeloablative pediatric blood and marrow transplantation. Biol. Blood Marrow Transpl. 19, 569–575. 10.1016/j.bbmt.2012.12.015 PMC4231793 23266742

[B21] Harbers V. E. M. Zwerink L. G. J. M. Rongen G. A. Klein W. M. van der Vleuten C. J. M. van Rijnsoever I. M. P. (2023). Clinical differences in sirolimus treatment with low target levels between children and adults with vascular malformations - a nationwide trial. Clin. Transl. Sci. 16, 781–796. 10.1111/cts.13488 36824030 PMC10176016

[B22] Hartinger J. M. Ryšánek P. Slanař O. Šíma M. (2022). Pharmacokinetic principles of dose adjustment of mTOR inhibitors in solid organ transplanted patients. J. Clin. Pharm. Ther. 47, 1362–1367. 10.1111/jcpt.13753 35934622

[B23] Holford N. Heo Y.-A. Anderson B. (2013). A pharmacokinetic standard for babies and adults. J. Pharm. Sci. 102, 2941–2952. 10.1002/jps.23574 23650116

[B24] Ji Y. Chen S. Yang K. Zhou J. Zhang X. Jiang X. (2021). A prospective multicenter study of sirolimus for complicated vascular anomalies. J. Vasc. Surg. 74, 1673–1681.e3. 10.1016/j.jvs.2021.04.071 34082006

[B25] Kahan B. D. Napoli K. L. Kelly P. A. Podbielski J. Hussein I. Urbauer D. L. (2000). Therapeutic drug monitoring of sirolimus: correlations with efficacy and toxicity. Clin. Transpl. 14, 97–109. 10.1034/j.1399-0012.2000.140201.x 10770413

[B26] Kardas P. Lewek P. Matyjaszczyk M. (2013). Determinants of patient adherence: a review of systematic reviews. Front. Pharmacol. 4, 91. 10.3389/fphar.2013.00091 23898295 PMC3722478

[B27] Kovarik J. M. Sabia H. D. Figueiredo J. Zimmermann H. Reynolds C. Dilzer S. C. (2001). Influence of hepatic impairment on everolimus pharmacokinetics: implications for dose adjustment. Clin. Pharmacol. Ther. 70, 425–430. 10.1016/s0009-9236(01)15633-x 11719728

[B28] Lackner H. Karastaneva A. Schwinger W. Benesch M. Sovinz P. Seidel M. (2015). Sirolimus for the treatment of children with various complicated vascular anomalies. Eur. J. Pediatr. 174, 1579–1584. 10.1007/s00431-015-2572-y 26040705

[B29] Lang J. Vincent L. Chenel M. Ogungbenro K. Galetin A. (2021). Impact of hepatic CYP3A4 ontogeny functions on drug-drug interaction risk in pediatric physiologically-based pharmacokinetic/pharmacodynamic modeling: critical literature review and ivabradine case study. Clin. Pharmacol. Ther. 109, 1618–1630. 10.1002/cpt.2134 33283268

[B30] Le Meur Y. Djebli N. Szelag J.-C. Hoizey G. Toupance O. Rérolle J. P. (2006). CYP3A5*3 influences sirolimus oral clearance in *de novo* and stable renal transplant recipients. Clin. Pharmacol. Ther. 80, 51–60. 10.1016/j.clpt.2006.03.012 16815317

[B31] Li S. Zhan M. Wu S. Liao J. Xu H. Sun D. (2022). Population pharmacokinetic analysis and dosing optimization of sirolimus in children with tuberous sclerosis complex. J. Clin. Pharmacol. 62, 948–959. 10.1002/jcph.2033 35094415

[B32] Maruani A. Tavernier E. Boccara O. Mazereeuw-Hautier J. Leducq S. Bessis D. (2021). Sirolimus (rapamycin) for slow-flow malformations in children: the observational-phase randomized clinical PERFORMUS trial. JAMA Dermatol. 157, 1289–1298. 10.1001/jamadermatol.2021.3459 34524406 PMC8444064

[B33] McCune J. S. Bemer M. J. Long-Boyle J. (2016). Pharmacokinetics, pharmacodynamics, and pharmacogenomics of immunosuppressants in allogeneic hematopoietic cell transplantation: Part II. Clin. Pharmacokinet. 55, 551–593. 10.1007/s40262-015-0340-9 26620047 PMC4824644

[B34] Methaneethorn J. Art-Arsa P. Kosiyaporn R. Leelakanok N. (2022). Predictors of sirolimus pharmacokinetic variability identified using a nonlinear mixed effects approach: a systematic review. J. Popul. Ther. Clin. Pharmacol. = J. de La Ther. Des Populations de La Pharmacol. Clinique 29, e11–e29. 10.47750/jptcp.2022.940 36308280

[B35] Mizuno T. Emoto C. Fukuda T. Hammill A. M. Adams D. M. Vinks A. A. (2017). Model-based precision dosing of sirolimus in pediatric patients with vascular anomalies. Eur. J. Pharm. Sci. 109, S124–S131. 10.1016/j.ejps.2017.05.037 28526601

[B36] Mizuno T. O’Brien M. M. Vinks A. A. (2019). Significant effect of infection and food intake on sirolimus pharmacokinetics and exposure in pediatric patients with acute lymphoblastic leukemia. Eur. J. Pharm. Sci. 128, 209–214. 10.1016/j.ejps.2018.12.004 30537529

[B37] Moes D. J. A. R. Guchelaar H.-J. de Fijter J. W. (2015). Sirolimus and everolimus in kidney transplantation. Drug Discov. Today 20, 1243–1249. 10.1016/j.drudis.2015.05.006 26050578

[B38] Mould D. R. Upton R. N. (2013). Basic concepts in population modeling, simulation, and model-based drug development-part 2: introduction to pharmacokinetic modeling methods. Cpt Pharmacometrics Syst. Pharmacol. 2, e38. 10.1038/psp.2013.14 23887688 PMC3636497

[B39] Ozeki M. Nozawa A. Yasue S. Endo S. Asada R. Hashimoto H. (2019). The impact of sirolimus therapy on lesion size, clinical symptoms, and quality of life of patients with lymphatic anomalies. Orphanet J. Rare Dis. 14, 141. 10.1186/s13023-019-1118-1 31196128 PMC6567608

[B40] Queisser A. Seront E. Boon L. M. Vikkula M. (2021). Genetic basis and therapies for vascular anomalies. Circ. Res. 129, 155–173. 10.1161/CIRCRESAHA.121.318145 34166070

[B41] Sabo A.-N. Jannier S. Becker G. Lessinger J.-M. Entz-Werlé N. Kemmel V. (2021). Sirolimus pharmacokinetics variability points to the relevance of therapeutic drug monitoring in pediatric oncology. Pharmaceutics 13, 470. 10.3390/pharmaceutics13040470 33808416 PMC8067051

[B42] Sandbank S. Molho-Pessach V. Farkas A. Barzilai A. Greenberger S. (2019). Oral and topical sirolimus for vascular anomalies: a multicentre study and review. Acta Derm. Venerol. 99, 990–996. 10.2340/00015555-3262 31304557

[B43] Schachter A. D. Benfield M. R. Wyatt R. J. Grimm P. C. Fennell R. S. Herrin J. T. (2006). Sirolimus pharmacokinetics in pediatric renal transplant recipients receiving calcineurin inhibitor co-therapy. Pediatr. Transpl. 10, 914–919. 10.1111/j.1399-3046.2006.00541.x PMC1636453 17096757

[B44] Schachter A. D. Meyers K. E. Spaneas L. D. Palmer J. A. Salmanullah M. Baluarte J. (2004). Short sirolimus half-life in pediatric renal transplant recipients on a calcineurin inhibitor-free protocol. Pediatr. Transpl. 8, 171–177. 10.1046/j.1399-3046.2003.00148.x PMC1350260 15049798

[B45] Seront E. Van Damme A. Legrand C. Bisdorff-Bresson A. Orcel P. Funck-Brentano T. (2023). Preliminary results of the European multicentric phase III trial regarding sirolimus in slow-flow vascular malformations. JCI Insight 8, e173095. 10.1172/jci.insight.173095 37937645 PMC10721262

[B46] Shao S. Hu L. Han Z. Hou K. Fang H. Zhang G. (2020). The effect of ABCB1 polymorphism on sirolimus in renal transplant recipients: a meta-analysis. Transl. Androl. Urology 9 (No 2), 673–683. 10.21037/tau.2020.03.42 PMC7215018 32420174

[B47] Shen G. Moua K. T. Y. Perkins K. Johnson D. Li A. Curtin P. (2023). Precision sirolimus dosing in children: the potential for model-informed dosing and novel drug monitoring. Front. Pharmacol. 14, 1126981. 10.3389/fphar.2023.1126981 37021042 PMC10069443

[B48] Stenton S. B. Partovi N. Ensom M. H. H. (2005). Sirolimus: the evidence for clinical pharmacokinetic monitoring. Clin. Pharmacokinet. 44, 769–786. 10.2165/00003088-200544080-00001 16029064

[B49] Stillo F. Mattassi R. Diociaiuti A. Neri I. Baraldini V. Dalmonte P. (2022). Guidelines for vascular anomalies by the Italian society for the study of vascular anomalies (SISAV). Int. Angiol. 41, 1–130. 10.23736/S0392-9590.22.04902-1 35546136

[B50] Wang D. Chen X. Li Z. (2019). Population pharmacokinetics of sirolimus in pediatric patients with kaposiform hemangioendothelioma: a retrospective study. Oncol. Lett. 18, 2412–2419. 10.3892/ol.2019.10562 31452734 PMC6676569

[B51] Wang D.-D. Chen X. Xu H. Li Z.-P. (2020). Initial dosage recommendation for sirolimus in children with tuberous sclerosis complex. Front. Pharmacol. 11, 890. 10.3389/fphar.2020.00890 32595509 PMC7300220

[B52] Werk A. N. Cascorbi I. (2014). Functional gene variants of CYP3A4. Clin. Pharmacol. Ther. 96, 340–348. 10.1038/clpt.2014.129 24926778

[B53] Wiegand S. Dietz A. Wichmann G. (2022). Efficacy of sirolimus in children with lymphatic malformations of the head and neck. Eur. Arch. Otorhinolaryngol. 279, 3801–3810. 10.1007/s00405-022-07378-8 35526176 PMC9249683

[B54] Woillard J.-B. Kamar N. Coste S. Rostaing L. Marquet P. Picard N. (2013). Effect of CYP3A4*22, POR*28, and PPARA rs4253728 on sirolimus *in vitro* metabolism and trough concentrations in kidney transplant recipients. Clin. Chem. 59, 1761–1769. 10.1373/clinchem.2013.204990 23974086

[B55] Zhang Y. Quan Y. Wang D. Cassady K. Zou W. Xiong J. (2023). Optimizing the therapeutic window of sirolimus by monitoring blood concentration for the treatment of immune thrombocytopenia. Platelets 34, 2277831. 10.1080/09537104.2023.2277831 38050853

[B56] Zhang Z. Li Y. Zhang G. Yang K. Qiu T. Zhou J. (2022). Safety evaluation of oral sirolimus in the treatment of childhood diseases: a systematic review. Child. Basel, Switz. 9, 1295. 10.3390/children9091295 PMC9497617 36138604

[B57] Zhao Y.-T. Dai H.-R. Li Y. Zhang Y.-Y. Guo H.-L. Ding X.-S. (2022). Comparison of LC-MS/MS and EMIT methods for the precise determination of blood sirolimus in children with vascular anomalies. Front. Pharmacol. 13, 925018. 10.3389/fphar.2022.925018 36147342 PMC9486013

[B58] Zimmerman J. J. Kahan B. D. (1997). Pharmacokinetics of sirolimus in stable renal transplant patients after multiple oral dose administration. J. Clin. Pharmacol. 37, 405–415. 10.1002/j.1552-4604.1997.tb04318.x 9156373

[B59] Zimmerman J. J. Patat A. Parks V. Moirand R. Matschke K. (2008). Pharmacokinetics of sirolimus (rapamycin) in subjects with severe hepatic impairment. J. Clin. Pharmacol. 48, 285–292. 10.1177/0091270007312902 18218785

