# Supplementary material for: Population pharmacokinetic study in children with vascular anomalies: body weight as a key variable in predicting the initial dose and dosing frequency of sirolimus
Source: Front Pharmacol. 2024 Sep 24;15:1457614. doi: 10.3389/fphar.2024.1457614 (PMC11458483; doi:10.3389/fphar.2024.1457614)
Supplement: Supplementary file 1 [file Table1.DOCX]

**Population pharmacokinetic study in children with vascular anomalies: Body weight as a key variable in predicting the initial dose and dosing frequency of sirolimus**

Lin Fan^1^, Hong-Li Guo^1^, Yue-Tao Zhao^1,2, †^, Yue Li^1^, Wei-Jun Wang^1,2, †^, Jian Huang^1^, Ya-Hui Hu^1, *^, Ji-Jun Zou^3, *^, Feng Chen^1, *^

^1^Pharmaceutical Sciences Research Center, Department of Pharmacy, Children's Hospital of Nanjing Medical University, Nanjing 210008, China.

^2^School of Basic Medicine and Clinical Pharmacy, China Pharmaceutical University, Nanjing 211198, China.

^3^Department of Burns and Plastic Surgery, Children's Hospital of Nanjing Medical University, Nanjing 210008, China

^†^Visiting graduate student from China Pharmaceutical University.

^*^Corresponding authors at Children’s Hospital of Nanjing Medical University, 72 Guangzhou Road, Nanjing 210008, China.

# E-mail addresses: huyahui324@163.com (Ya-Hui Hu), 5098782@qq.com (J.J. Zou), cy.chen508@gmail.com (Feng Chen).

**----Supplemental Material---**

**Table S1** Summary of population pharmacokinetic studies on sirolimus in children with vascular anomalies

| Study | Indication | Country | Sampling strategy | number of subjects (male/female) | number of observations | Age (year) Mean **±** SD [Range] | Weight (kg) Mean **±** SD [Range] | Pharmacokinetic parameters |
| --- | --- | --- | --- | --- | --- | --- | --- | --- |
| Chen  (2021) | Lymphangioma | China | NR | 15 (12/3) | NR | 7.29±3.11  [0.12-16.39] | 22.27±9.87  [4-54] | CL/F=11.3×(BW/70)^0.75^  V/F=388×(BW/70)  Ka=0.485 (fixed) |
| Chen  (2020) | Kaposiform haemangioendothelioma | China | C_trough_ | 14 (9/5) | NR | 1.53±1.40 | 8.87±4.12 | CL/F=7.55×(BW/70)^0.75^× (1+0.999×CYP3A5) (CYP3A5=0 if CYP3A5*3/*3; CYP3A5=1 if CYP3A5*1)  V/F=1840×(BW/70)  Ka=0.485 (fixed) |
| Wang (2019) | Kaposiform hemangioendothelioma | China | C_trough_ | 17 (11/6) | 133 | 1.21±1.20  [0.2-6] | 7.99±3.04  [3.6-18] | CL/F=3.19×e^0.215AGE^×e^0.0108ALT^×e^-0.818sex^  V/F=165×e^0.0783DTT/10^  Ka=0.485 (fixed) |
| Mizuno  (2017) | Vascular anomalies | USA | C_trough_ | 52 (20/32) | 676 | 4.9^a^ [0.1-18.6] | 18.4 ^a^ [4-100.6] | CL/F=18.5×(BW/70)^0.67^×PMA^2.94^/(62.9^2.94^+PMA^2.94^)  V/F=1030×(BW/70）  Ka=2.77 (fixed) |
| Our study | Vascular anomalies | China | C_trough_ | 49 (24/25) | 134 | 4.15±3.1  [0.08-12] | 17.88±9.66  [3.3-65] | CL/F=4.06×(BW/16)^1.23^  V/F=155×(BW/16)^1.62^  Ka=0.485 (fixed) |

Abbreviations: K_a_, absorption rate constant; CL, clearance; V_d_, apparent volume of distribution; BW, body weight; ALT, alanine transaminase; DTT, duration of treatment with sirolimus; PMA, postmenstrual age.

^a^ presented as median value

**Table S2** Genotyping information for candidate single nucleotide polymorphisms (SNPs)

| **Gene** | **SNPs** | **1st-PCRP** | **2nd-PCRP** | **Amplification length (bp)** | **UEP_SEQ** |
| --- | --- | --- | --- | --- | --- |
| *ABCB1* | rs1045642 | ACGTTGGATGAAGGCATGTATGTTGGCCTC | ACGTTGGATGTTGCCTATGGAGACAACAGC | 99 | CCTTTGCTGCCCTCAC |
| *ABCB1* | rs1128503 | ACGTTGGATGTTTCTCACTCGTCCTGGTAG | ACGTTGGATGCACAGCCACTGTTTCCAACC | 110 | aGTCCTGGTAGA TCTTGAAGGG |
| *ABCB1* | rs2032582 | ACGTTGGATGCATATTTAGTTTGACTCACC | ACGTTGGATGTGTTGTCTGGACAAGCACTG | 92 | ccacTTAGTTTGACTCACCTTCCCAG |
| *ABCC2* | rs3740066 | ACGTTGGATGGTCCTCAGAGGGATCACTTG | ACGTTGGATGTGTTTGATCACAAGGCCTCC | 110 | AGGGATCACTTGTGACAT |
| *ABCC2* | rs717620 | ACGTTGGATGAGCATGATTCCTGGACTGCG | ACGTTGGATGCCTGTTCCACTTTCTTTGATG | 117 | ccccGACTGCGTCTGGAAC |
| *CYP3A4* | rs4646437 | ACGTTGGATGCTTCAAAAGATGCACAAGGG | ACGTTGGATGAGGGCAGGTCTATGCATAAG | 90 | TGCTGATCTCACTGCTGTAG |
| *CYP3A4* | rs2242480 | ACGTTGGATGTGCTAAGGTTTCACCTCCTC | ACGTTGGATGGCAGGAGGAAATTGATGCAG | 102 | aaCCCTCCTTCTCCATGTA |
| *CYP3A5* | rs776746 | ACGTTGGATGACCCAGCTTAACGAATGCTC | ACGTTGGATGGTAATGTGGTCCAAACAGGG | 118 | aaggAGAGCTCTTTTGTCTTTCA |
| *CYP3A7* | rs12360 | ACGTTGGATGGAAATCTCTGGTGTTCTGGG | ACGTTGGATGGGAACCCTAAGTGGAGAATG | 104 | gggcaGGGGCACAGCTTTCTT |
| *CYP3A7* | rs10211 | ACGTTGGATGTACAGACCATGAGAGAGCAC | ACGTTGGATGAATGAAGACGGGCTTCATCC | 100 | gatCACAATGCACGTACAGAATCCC |
| *CYP3A7* | rs2257401 | ACGTTGGATGGTGGTGGTGATGATTCCAAG | ACGTTGGATGCTTTCAGGGAGGAACTTCTC | 100 | gagtaATCATGACCCAAAGTACTGGA |
| *mTOR* | rs1770345 | ACGTTGGATGGGTTAAATCAGACAAGCCAC | ACGTTGGATGAACTTAGCCTTAGGCATTGG | 111 | gTTGTGGAATATTATCTCTCTCT |
| *mTOR* | rs1883965 | ACGTTGGATGATTGTGTATCTCTGCCCCAG | ACGTTGGA TGCTGGA TCTCTGAGAGGTGTC | 101 | TGCCCCAGGGAACAC |
| *mTOR* | rs2076655 | ACGTTGGATGAACCAACCCATCATGTACTG | ACGTTGGATGCCAATGAATAGCCTCTCACC | 116 | GCCAAAAACCGTTTACT |
| *mTOR* | rs2300095 | ACGTTGGATGCACCCTGGGCAAATTACTTG | ACGTTGGATGCGGCTAAGCCATTTAGACAC | 102 | tctaCTCAGAGTGCCTCATCTA |
| POR | rs1057868 | ACGTTGGATGACTTGCGCACGAACATGGG | ACGTTGGATGAACAAGGGCGTGGCCACCAA | 111 | tttctGCCGCCGTTCTCCCCG |
| IL10 | rs1800896 | ACGTTGGATGATTCCATGGAGGCTGGATAG | ACGTTGGATGGACAACACTACTAAGGCTTC | 107 | ggtgCCTATCCCTACTTCCCC |
| IL18 | rs5744247 | ACGTTGGATGGAACCCACTGGTTGTAAGAG | ACGTTGGATGCCTGAGGATGCCATAAACAC | 99 | TGTAAGAGAAGGATGAAGC |
| SUMO4 | rs237024 | ACGTTGGATGTGCTTGTGTACACATACCAG | ACGTTGGATGTTCTTTTGTTTCCCCCTTCC | 86 | CATACCAGTTACTTCATGTATAATAAA |
| NR1I2 | rs3814055 | ACGTTGGATGTCACCTGAAGACAACTGTGG | ACGTTGGATGGAGACCACGATTGAGCAAAC | 96 | TTTGGCAATCCCAGGTT |
| NR1I2 | rs6785049 | ACGTTGGATGACAGTCATCCTCAGGGAAAG | ACGTTGGATGGCCATCCCATAATCCAGAAG | 91 | CCTCCCTCTTCCTCTC |
| TCF7L2 | rs7903146 | ACGTTGGATGAACTAAGGGTGCCTCATACG | ACGTTGGATGGCCTCAAAACCTAGCACAGC | 120 | GTGCCTCATACGGCAATTAAATTATATA |

**Table S3** Complete demographic and clinical characteristics of enrolled subjects

| **Characteristic** | **Median (range)** |
| --- | --- |
| **Demographic** |  |
| Sex, male/female |  |
| Age, y | 3.5 (0.08 - 12) |
| WT, kg | 16 (3.3 - 65) |
| **Laboratory parameter** |  |
| RBC (1012/L) | 4.64 (2.73 - 5.9) |
| WBC (109/L) | 8 (4.33 - 17.14) |
| HGB (g/L) | 122 (77 - 172) |
| MCH (pg) | 26.7 (19.4 - 30.6) |
| MCHC (g/L) | 330 (213 - 361) |
| HCT (%) | 36.8 (24.2 - 49.4) |
| ALB (g/L) | 44.8 (37.2 - 52.2) |
| ALT (U/L) | 12 (5 - 34) |
| AST (U/L) | 27 (17 - 52) |
| HDL (mmol/L) | 1.38 (0.6 - 2.15) |
| TBIL (μmol/L) | 5.5 (1.6 - 16) |
| DBIL (μmol/L) | 1.97 (0 - 7.3) |
| SCR (μmol/L) | 27 (13 - 53) |
| BUN (mmol/L) | 4.31 (0.9 - 7.7) |
| UA (μmol/L) | 210 (72 - 403) |
| CYS (mg/L) | 0.97 (0.58 - 1.79) |
| **Genotype** |  |
| ***ABCB1*** |  |
| rs1128503 (%), AA/GA/GG | 27/15/7 |
| ***ABCC2*** |  |
| rs717620 (%), CC/CT/TT | 34/14/1 |
| ***CYP3A4*** |  |
| rs4646437 (%), GG/GA/AA | 34/12/3 |
| rs2242480 (%), CC/CT/TT | 28/15/6 |
| ***CYP3A5*** |  |
| rs776746 (%), TT/CT/CC | 5/22/22 |
| ***CYP3A7*** |  |
| rs12360 (%), AA/GA/GG | 17/16/16 |
| rs10211 (%), TT/CT/CC | 21/23/5 |
| rs2257401 (%), GG/GC/CC | 23/21/5 |
| ***mTOR*** |  |
| rs1883965 (%), AG/GG | 7/42 |
| rs2076655 (%), GG/GA/AA | 3/15/31 |
| rs2300095 (%), GA/GG | 7/42 |
| ***POR*** |  |
| rs1057868 (%), CC/CT/TT | 21/24/4 |
| ***IL10*** |  |
| rs1800896 (%), TT/CC | 39/10 |
| ***IL18*** |  |
| rs5744247 (%), GG/GC/CC | 21/18/10 |
| ***SUMO4*** |  |
| rs237024 (%), TT/TC/CC | 2/19/28 |
| ***NR1I2*** |  |
| rs3814055 (%), CC/CT/TT | 25/22/2 |
| rs6785049 (%), GG/GA/AA | 18/19/12 |
| ***TCF7L2*** |  |
| rs7903146 (%), CC/TC | 48/1 |

Abbreviations: BW, total body weight; RBC, red blood cell count; WBC, white blood cell count; HGB, hemoglobin; MCH, mean corpuscular hemoglobin; MCHC, mean corpuscular hemoglobin concentration; HCT, hematocrit; ALB, albumin; ALT, alanine aminotransferase; AST, aspartate amino-transferase; HDL, high-density lipoprotein cholesterol; TBIL, total bilirubin; DBIL, direct bilirubin; SCR, serum creatinine; BUN, blood urea nitrogen; UA, uric acid; CYSC, cystatin-C.

**Table S4** The establishing process of model

| **Model description** | **Equation** | **OFV** | **ΔOFV** | **Reference model** | **Reserve** |
| --- | --- | --- | --- | --- | --- |
| Base model: one-compartment mode with first-order elimination | 1, 2 | 637.093 | - | - | - |
| Size and maturation model | | | | |  |
| **Model I: Simple exponent model** | **6, 7** | **522.876** | **-114.217** | **Base model** | **Yes** |
| Model II: fixed allometric exponent model | 8, 9 | 552.133 | - 84.960 | Base model | No |
| Model III: sigmoid maturation model | 10 | 816.308 | 179.845 | Base model | No |
| Model IV: Weight dependent exponent model | 11 | 527.857 | - 109.234 | Base model | No |
| Model V: Age dependent exponent model | 12 | 527.857 | - 109.234 | Base model | No |
| Stepwise forward inclusion-Round 1 | | | | | |
| Model 1: Add SEX on CL | 17 | 542.924 | 20.048 | Model I | No |
| Model 2: Add RBC on CL | 14 | 522.178 | - 0.698 | Model I | No |
| Model 3: Add WBC on CL | 14 | 515.939 | - 6.937 | Model I | No |
| Model 4: Add HB on CL | 15 | 521.579 | - 1.297 | Model I | No |
| Model 5: Add MCH on CL | 14 | 517.342 | - 5.534 | Model I | No |
| Model 6: Add MCHC on CL | 14 | 521.27 | - 1.606 | Model I | No |
| Model 7: Add HCT on CL | 13 | 516.986 | - 5.890 | Model I | No |
| Model 8: Add ALB on CL | 14 | 522.843 | -0.033 | Model I | No |
| Model 9: Add ALT on CL | 15 | 521.214 | -1.662 | Model I | No |
| Model 10: Add AST on CL | 15 | 518.76 | - 4.116 | Model I | No |
| Model 11: Add HDL on CL | 13 | 520.662 | -2.214 | Model I | No |
| Model 12: Add TBIL on CL | 13 | 519.298 | - 3.578 | Model I | No |
| Model 13: Add DBIL on CL | 13 | 520.049 | -2.827 | Model I | No |
| Model 14: Add BUN on CL | 15 | 517.009 | -5.867 | Model I | No |
| Model 15: Add SCR on CL | 13 | 521.764 | - 1.112 | Model I | No |
| **Model 16: Add UA on CL** | **13** | **513.96** | **- 8.916** | **Model I** | **Yes** |
| Model 17: Add CYS on CL | 14 | 522.579 | - 0.297 | Model I | No |
| Model 18: Add rs776746 on CL | 17 | 522.533 | - 0.343 | Model I | No |
| Model 19: Add rs10211 on CL | 17 | 522.284 | - 0.592 | Model I | No |
| Model 20: Add rs1057868 on CL | 17 | 522.719 | - 0.157 | Model I | No |
| Model 21: Add rs1128503 on CL | 16 | 522.15 | - 0.726 | Model I | No |
| Model 22: Add rs12360 on CL | 17 | 522.693 | - 0.183 | Model I | No |
| Model 23: Add rs1800896 on CL | 17 | 521.371 | - 1.505 | Model I | No |
| Model 24: Add rs1883965 on CL | 17 | 521.121 | - 1.755 | Model I | No |
| Model 25: Add rs2076655 on CL | 17 | 522.851 | - 0.025 | Model I | No |
| Model 26: Add rs2257401 on CL | 16 | 520.048 | - 2.828 | Model I | No |
| Model 27: Add rs2300095 on CL | 17 | 521.121 | - 1.755 | Model I | No |
| Model 28: Add rs237024 on CL | 17 | 522.827 | - 0.049 | Model I | No |
| Model 29: Add rs3814055 on CL | 16 | 521.116 | - 1.760 | Model I | No |
| Model 30: Add rs4646437 on CL | 16 | 520.136 | - 2.740 | Model I | No |
| Model 31: Add rs5744247 on CL | 16 | 514.195 | - 8.681 | Model I | No |
| Model 32: Add rs6785049 on CL | 17 | 521.576 | - 1.300 | Model I | No |
| Model 33: Add rs717620 on CL | 16 | 519.508 | - 3.368 | Model I | No |
| Model 34: Add rs7903146 on CL | 17 | 522.423 | - 0.453 | Model I | No |
| Stepwise forward inclusion-Round 2 | | | | | |
| Model 35: Add rs5744247 on CL | 17 | 510.294 | - 3.701 | Model 16 | No |
| Model 36: Add WBC on CL | 15 | 510.904 | -3.056 | Model 16 | No |
| Model 37: Add MCH on CL | 14 | 510.233 | - 3.727 | Model 16 | No |
| Model 38: Add HCT on CL | 13 | 513.897 | -0.063 | Model 16 | No |
| Model 39: Add BUN on CL | 15 | 510.149 | -3.811 | Model 16 | No |
| Model 40: Add AST on CL | 13 | 511.027 | -2.933 | Model 16 | No |
| Stepwise backward exclusion-Round 1 | | | | | |
| Model 41: Remove UA on CL | 13 | 522.876 | 8.916 | Model 16 | No |

Abbreviations: ΔOFV, change in objective function value; BW, total body weight; RBC, red blood cell count; WBC, white blood cell count; HGB, hemoglobin; MCH, mean corpuscular hemoglobin; MCHC, mean corpuscular hemoglobin concentration; HCT, hematocrit; ALB, albumin; ALT, alanine aminotransferase; AST, aspartate amino-transferase; HDL, high-density lipoprotein cholesterol; TBIL, total bilirubin; DBIL, direct bilirubin; SCR, serum creatinine; BUN, blood urea nitrogen; UA, uric acid; CYSC, cystatin-C.

**Table S5** NPC result of the final model (1000 simulations)

| **PI** | **Count (%) of DV below PI** | **95% CI of DV below PI** | **Count (%) of DV above PI** | **95% CI of DV above PI** |
| --- | --- | --- | --- | --- |
| 0% | 79 (58.96) | 39.55-61.19 | 55 (41.04) | 38.81-60.45 |
| 20% | 60 (44.78) | 29.85-50.75 | 45 (33.68) | 29.85-50.75 |
| 40% | 46 (34.33) | 20.90-40.30 | 39 (29.10) | 20.15-40.30 |
| 50% | 36 (26.87) | 16.42-35.07 | 31 (23.13) | 14.93-34.33 |
| 60% | 25 (18.66) | 11.94-29.10 | 23 (17.16) | 11.19-29.10 |
| 80% | 9 (6.72) | 4.48-16.42 | 15 (11.19) | 4.48-17.16 |
| 90% | 2 (1.49) | 1.49-9.70 | 8 (5.97) | 0.75-9.70 |
| 95% | 2 (1.49) | 0.00-5.97 | 4 (2.99) | 0.00-5.97 |

Abbreviations: CI, confidence interval; DV, dependent variable; NPC, numerical predictive check; PI, prediction interval.
